# Prevalence of Temporomandibular Joint Disorders Among Students of the University of Jordan

**DOI:** 10.4021/jocmr2009.06.1245

**Published:** 2009-07-03

**Authors:** Soukaina Ryalat, Zaid H Baqain, Wala M. Amin, Faleh Sawair, Osama Samara, Darwish H. Badran

**Affiliations:** a Faculty of Dentistry, University of Jordan, Jordan; b Faculty of Medicine, University of Jordan, Jordan

## Abstract

**Background:**

This study aimed to investigate the prevalence of temporomandibular disorder (TMD) among students of the University of Jordan.

**Methods:**

Information about the symptoms of TMD and the possible risk factors were collected using specifically designed questionnaires. The collected data sets were treated statistically using the SPSS release 14 package.

**Results:**

The results of the present investigation showed that pain in or about the ears or cheeks was the most prevalent symptom whereas locking of the temporomandibular joint (TMJ) was the least prevalent. Nearly one-third of the investigated sample (31.4%, 346/1103) had no symptoms of TMD whereas 68.6% (757/1103) had at least one symptom. Students of health science studies had significantly the highest risk in developing TMJ clicking compared to students studying pure science or humanitarian studies.

**Conclusions:**

TMD is of a high prevalence among students of the University of Jordan, particularly among students of health and science studies, which signify the role of stress in the development and/or progression of TMD. The findings of this study are alarming and entailing further investigations to identify risk factors associated with TMD in order to establish measures for prevention and treatment.

**Keywords:**

TMD; Clicking; Health sciences; Humanitarian studies

## Introduction

Temporomandibular joint disorder (TMD) represents a common health problem [1]. It is an umbrella term embracing a number of clinical manifestations that involve the temporomandibular joint (TMJ), the masticatory muscles and the teeth. Patients with TMD usually suffer from muscle and/or joint pain on palpation and on mandibular movements, joint sounds and the mandibular range of motion may be limited [2]. TMD can affect any patients regardless of age including children [3] or gender with varying signs and symptoms [4]. However, due to the variation in symptoms among different patients and in the same patient at different times, the diagnosis of this clinical entity may be difficult [5].

The prevalence of TMD in the general population is high [6], between 40% to 60% [7]. One study reported that 87% of a sample of 1040 subjects had one or more positive symptoms or clinical signs of TMD [4]. Individuals with low self esteem are more likely to suffer from TMD [8], psychological and emotional factors are clearly involved in the development of the disorder [9, 10].

Questionnaires are usually used to gather information about the prevalence of TMD in the population. The objectives of the present investigation were: to study the prevalence of TMD among students at the University of Jordan, using the guidelines recommended by the American Dental Association in 1982 [4], and secondly,  to compare the prevalence of TMD among students of different faculties in an attempt to recognize the risk factors for TMD development. It is hoped that the findings of the present study would serve as baseline data for future investigations in this field.

## Subjects and Methods

The study sample consisted of 1103 students studying at the University of Jordan. There were 276 males and 827 females and their age range was between 18 and 25 years old. The sample comprised 353 (32.0%) first year students; 548 (49.7%) second year students; 168 (15.2%) third year students; and 34 (3.1%) fourth year students. Of the 1103 students, 482 (43.7%) were studying in humanitarian colleges; 163 (14.8%) were studying in science colleges; and 458 (41.5%) were studying in health colleges.

A questionnaire was composed and distributed randomly to students belonging to faculties of health, science and humanitarian studies. The completed questionnaire contained the following items regarding the different symptoms of TMD, and the possible risk factors:

Do you hear joint sounds?
Do you have limitation in mouth opening?
Do you have pain in or about the ears?
Have you ever had joint locking? If yes, how many times?
Do you have pain on chewing?
Have you ever had trauma to head and neck area? If yes, how many times?
Do you have stress, or under stressful conditions?
Do you have arthralgia in other joints in your body?
Do you live with your family?

Students were asked to check items that were most relevant to their conditions.

Statistical analysis was performed using SPSS for Windows release 14.0 (SPSS Inc., Chicago, IL, USA). Chi-square tests were used to determine the associations between the TMD symptoms (clicking, trismus, pain in or about the ears, TMJ locking and pain on chewing or yawing) and independent factors. Stepwise multivariate logistic regression was then used to control for potential confounding variables and to calculate the odd ratios (ORs) for potential independent variables for the TMD symptoms. Differences at the 5% level were accepted as significant.

## Results

**Figure 1:**
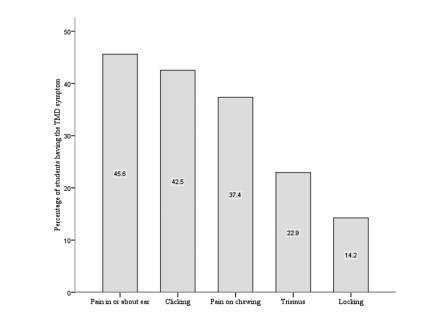
A histogram showing the prevalence of TMD symptoms in the investigated sample.

**Figure 2:**
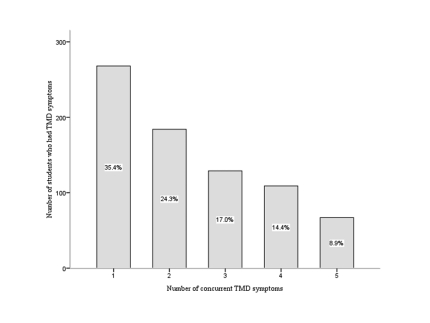
Number of students who had concurrent TMD symptoms.

The prevalence of various symptoms of TMD in the study population is shown in Figure 1. The most frequently reported TMD symptom in this study was pain in or about the ears or cheeks followed by clicking, and the least common was TMJ locking (Table 1). Nearly one-third of the students (31.4%, 346/1103) had no symptoms of TMD and 68.6% (757/1103) had at least one symptom. Of the 757 students who had TMD symptoms, the prevalence ranged from 35.4% (268 students) who had only one symptom to 8.9% (67 students) who had five concurrent TMD symptoms (Fig. 2). Significant associations were found between the five symptoms of TMD.

**Table 1:** Factors associated with TMD symptoms in the study sample

Factor		Clicking			Trismus			Pain about the ear			Locking			Pain on chewing		
		No.	%	P*	No.	%	P*	No.	%	P*	No.	%	P*	No.	%	P*
Age/years	18-19	139	39.4	0.14	68	19.3	0.18	166	47.0	0.23	51	14.4	0.28	128	36.3	0.16
	20-21	235	42.9		137	25.0		258	47.1		85	15.5		215	39.2	
	22-23	83	49.4		42	25.0		65	38.7		19	11.3		62	36.9	
	24-25	12	35.3		6	17.6		14	41.2		2	5.9		7	20.6	
Gender	Male	122	44.2	0.51	58	21.0	0.38	96	34.8	<0.01	42	15.2	0.59	103	37.3	0.99
	Female	347	42.0		195	23.6		407	49.2		115	13.9		309	37.4	
College	Humanity	169	35.1	<0.01	91	18.9	<0.01	231	47.9	<0.01	64	13.3	<0.01	164	34.0	<0.01
	Science	73	44.8		51	31.3		94	57.7		39	23.9		90	55.2	
	Health	227	49.6		111	24.2		178	38.9		54	11.8		158	34.5	
Trauma	No	412	41.3	0.01	210	21.0	<0.01	435	43.6	<0.01	123	12.3	<0.01	346	34.7	<0.01
	Yes	57	54.3		43	41.0		68	64.8		34	32.4		66	62.9	
Arthralgia	No	314	38.7	<0.01	154	19.0	<0.01	312	38.5	<0.01	92	11.3	<0.01	250	30.8	<0.01
	Yes	155	53.1		99	33.9		191	65.4		65	22.3		162	55.5	
Stress	No	178	33.1	<0.01	84	15.6	<0.01	190	35.4	<0.01	39	7.3	<0.01	132	24.6	<0.01
	Yes	291	51.4		169	29.9		313	55.3		118	20.8		280	49.5	
Family**	No	70	42.5	0.47	33	21.4	0.63	56	36.4	0.01	24	15.6	0.6	61	39.6	0.53
	Yes	399	42.0		220	23.2		447	47.1		133	14.0			37.0	

* P value of Chi square, ** Living with family

Factors associated with TMD symptoms in the study sample are shown in Table 1. The prevalence of TMD symptoms in the study population was not affected by age. Significantly more females than males had pain in or about the ears. Clicking was significantly highest in students of health colleges while trismus, pain in or about the ears, TMJ locking and pain on chewing were significantly highest in students of the science colleges. Stress was more common among students of health colleges (56.8%) than humanitarian colleges (44.8%) (P = 0.001) but comparable to those in the science colleges (55.2%). All the TMD symptoms were significantly more prevalent in students who had stress, positive history of trauma to the jaw, head, or neck, or positive history of arthralgia of other joints. Pain in or around the ears was significantly more prevalent in students who were living with their families.

Multivariate regression analysis for the possible factors associated with the various TMD symptoms is shown in Table 2. Students studying in health colleges had significantly the highest risk in developing TMJ clicking: 1.86 times greater risk compared with students studying in humanitarian colleges. However, those studying in science colleges had the highest risk in developing the other symptoms: 1.88, 1.53, 1.94, and 2.36 times greater risk to develop trismus, pain in or about the ears, TMJ locking, and pain on chewing or yawing, respectively compared with students studying in humanitarian colleges. Students who reported positive history of trauma to the jaw, head or neck region had significantly greater risk in developing all TMD symptoms. The risk ranged from one and a half times greater risk in developing clicking to three times greater risk in developing TMJ locking. Positive history of arthralgia in other joints was significantly associated with greater risk to develop clicking (OR = 1.56), trismus (OR = 1.76), pain in or about the ears and pain on chewing (OR = ≈ 2), but it was not significantly associated with the risk in developing TMJ locking. The risk to develop any of the TMD symptoms was significantly higher in students who had stress: the risk was two to three times greater than those with no stress. Gender had only significant risk in developing pain in or about the ears: females had nearly two times higher risk than male subjects. In contrast to the results of the univariate analysis, in multivariate analysis, living with family or alone was not a significant independent variable in the risk of developing pain in or about the ears.

**Table 2:** Stepwise logistic regression modelling for factors associated with TMD symptoms

TMD symptom	Variable	Regression Coefficient	P-value	OR	Confidence limits for OR
Clicking	College type		<0.01		
	Humanity	Reference category		1.00	
	Science	0.349	0.06	1.42	0.98-2.05
	Health	0.622	<0.01	1.86	1.42-2.45
	Positive history of trauma	0.486	0.02	1.63	1.07-2.47
	Arthralgia in other joints	0.442	<0.01	1.56	1.16-2.08
	Stress	0.579	<0.01	1.78	1.38-2.31

Trismus	College type		<0.01		
	Humanity	Reference category		1.00	
	Science	0.630	<0.01	1.88	1.24-2.85
	Health	0.374	0.03	1.45	1.05-2.02
	Positive history of trauma	0.872	<0.01	2.39	1.55-3.69
	Arthralgia in other joints	0.562	<0.01	1.76	1.27-2.42
	Stress	0.617	<0.01	1.85	1.36-2.53

Pain in or about ears	College type		<0.01		
	Humanity	Reference category		1.00	
	Science	0.424	0.03	1.53	1.04-2.24
	Health	-0.273	0.06	0.76	0.58-1.01
	Positive history of trauma	0.734	<0.01	2.08	1.34-3.25
	Arthralgia in other joints	0.822	<0.01	2.28	1.69-3.07
	Stress	0.647	<0.01	1.91	1.47-2.49
	Males	0.632	<0.01	1.88	1.39-2.55

Locking	College type		<0.01		
	Humanity	Reference category		1.00	
	Science	0.664	<0.01	1.94	1.22-3.09
	Health	-0.204	0.32	0.82	0.55-1.22
	Positive history of trauma	1.125	<0.01	3.08	1.93-4.92
	Stress	1.196	<0.01	3.31	2.23-4.89

Pain on chewing	College type		<0.01		
	Humanity	Reference category		1.00	
	Science	0.860	<0.01	2.36	1.61-3.47
	Health	0.039	0.79	1.04	0.78-1.39
	Positive history of trauma	1.038	<0.01	2.82	1.82-4.39
	Arthralgia in other joints	0.696	<0.01	2.01	1.49-2.70
	Stress	0.916	<0.01	2.5	1.90-3.29

## Discussion

The results of the present investigation showed that symptoms of TMD were remarkably prevalent among the 1103 students sample representative of the student community at the University of Jordan. This is in agreement with results reported on a sample of university students in Brazil [11]. It is accepted that the prevalence of TMD symptoms among university students, 18-25 years old, is higher than among older subjects [12], also higher than populations of rural areas [13].

This study demonstrated that pain in or around the ears was the most common symptom in the sample investigated, which was observed in a previous study [14]. Clicking was the second most common symptom, whereas, other studies demonstrated this to be the most common symptom [7, 15, 16]. It is possible that students confuse ear problems and TMD symptoms, finding it easier to express the joint pain as earache. This is further confirmed when it was stated that 50% of TMD patients were reviewed by otolaryngologists [17].

The results of the present study indicated that age variations within the investigated student sample had no significant effect on TMD symptoms. This finding supported a previous study [18] that investigated the age effect on TMD on a large sample of 7008 subjects, but contrasted the findings of other studies which reported either an increase in symptoms with age in a sample of 2255 subjects [19] or a decrease with age in a sample of 920 subjects [20]. The disagreement among the previously reported studies may be related to sample size or its demographic distribution.

It is accepted that TMD symptoms are more common in females [21, 22]. The findings of the present study pointed to some significant differences in the prevalence of TMD symptoms between the two sexes, with the females showing a higher prevalence of pain in or around the ears than males, but there was no difference in other symptoms, which is in accordance with previous studies [23, 24].

Trauma had a significant effect on developing TMD among the investigated sample, this is similar to previous findings which linked head and neck trauma with TMD symptoms, mainly as: joint pain, limitation of mouth opening and masticatory muscle tenderness [25-27].

Students in the health and science colleges had a significantly higher risk of developing TMD. A possible explanation is that the curricula of the health and science colleges entailed a greater study load; also admission to these colleges is more competitive than their humanitarian counterparts. It is likely that students in the former colleges are under greater stress and general anxiety. This study demonstrated a significant relationship between psychological and emotional factors and TMD. This finding is in agreement with those of previous reports [21, 28-30] which arrived at similar conclusion. Students in health colleges were at a significantly higher risk of developing clicking, whereas pain around the ears, joint locking and trismus were more common among students of science colleges. A possible explanation is that students of the health colleges are more aware than others of the commonality of the TMJ click in the population and its relationship to TMD, thus they would seek an early treatment of significant symptoms like trismus, joint locking, and pain on chewing and in or about the ears. Moreover, the inherent awareness of the health science students makes them conscientious to health matters and to a relatively more scrupulous attention to preventive issues and practices.

In conclusion, within the limitations of this study, the following conclusions may be drawn: first, TMD is of a high prevalence among students of the University of Jordan; second, TMD and its associated symptoms are frequent among students of health and science studies, which signify the role of stress in the development and\or progression of TMD.

More studies are required to identify risk factors associated with TMD to establish measures for prevention and treatment.

## References

[1] Sari S, Sonmez H (2002). Investigation of the relationship between oral parafunctions and temporomandibular joint dysfunction in Turkish children with mixed and permanent dentition. J Oral Rehabil.

[2] Schmitter M, Rammelsberg P, Hassel A (2005). The prevalence of signs and symptoms of temporomandibular disorders in very old subjects. J Oral Rehabil.

[3] Nilner M, Lassing SA (1981). Prevalence of functional disturbances and diseases of the stomatognathic system in 7-14 year olds. Swed Dent J.

[4] Nassif NJ, Hilsen KL (1992). Screening for temporomandibular disorders: history and clinical examination. American Dental Association. J Prosthodont.

[5] Cooper BC, Kleinberg I (2007). Examination of a large patient population for the presence of symptoms and signs of temporomandibular disorders. Cranio.

[6] Chuang SY (2002). Incidence of temporomandibular disorders (TMDs) in senior dental students in Taiwan. J Oral Rehabil.

[7] Okeson JP Management of Temporomandibular Disorders and Occlusion (ed 3). St. Louis.

[8] Godoy F, Rosenblatt A, Godoy-Bezerra J (2007). Temporomandibular disorders and associated factors in Brazilian teenagers: a cross-sectional study. Int J Prosthodont.

[9] Gerke DC, Goss AN, Bassett DL (1990). Psychological factors in temporomandibular joint dysfunction: life events. Aust Prosthodont J.

[10] Manfredini D, Landi N, Bandettini Di, Dell'Osso L, Bosco M (2003). A critical review on the importance of psychological factors in temporomandibular disorders. Minerva Stomatol.

[11] Pedroni CR, De Oliveira, Guaratini MI (2003). Prevalence study of signs and symptoms of temporomandibular disorders in university students. J Oral Rehabil.

[12] Osterberg T, Carlsson GE, Wedel A, Johansson U (1992). A cross-sectional and longitudinal study of craniomandibular dysfunction in an elderly population. J Craniomandib Disord.

[13] Goddard G, Karibe H (2002). TMD prevalence in rural and urban Native American populations. Cranio.

[14] Abdel-Hakim AM (1983). Stomatognathic dysfunction in the western desert of Egypt: an epidemiological survey. J Oral Rehabil.

[15] Jagger RG, Wood C (1992). Signs and symptoms of temporomandibular joint dysfunction in a Saudi Arabian population. J Oral Rehabil.

[16] Shiau YY, Chang C (1992). An epidemiological study of temporomandibular disorders in university students of Taiwan. Community Dent Oral Epidemiol.

[17] Abou-Atme YS, Zawawi KH, Melis M (2006). Prevalence, intensity, and correlation of different TMJ symptoms in Lebanese and Italian subpopulations. J Contemp Dent Pract.

[18] Gesch D, Bernhardt O, Alte D, Schwahn C, Kocher T, John U, Hensel E (2004). Prevalence of signs and symptoms of temporomandibular disorders in an urban and rural German population: results of a population-based Study of Health in Pomerania. Quintessence Int.

[19] Nilsson IM, List T, Drangsholt M (2007). Incidence and temporal patterns of temporomandibular disorder pain among Swedish adolescents. J Orofac Pain.

[20] Salonen L, Hellden L, Carlsson GE (1990). Prevalence of signs and symptoms of dysfunction in the masticatory system: an epidemiologic study in an adult Swedish population. J Craniomandib Disord.

[21] Conti PC, Ferreira PM, Pegoraro LF, Conti JV, Salvador MC (1996). A cross-sectional study of prevalence and etiology of signs and symptoms of temporomandibular disorders in high school and university students. J Orofac Pain.

[22] Poveda Roda R, Bagan JV, Diaz Fernandez JM, Hernandez Bazan S, Jimenez Soriano Y (2007). Review of temporomandibular joint pathology. Part I: classification, epidemiology and risk factors. Med Oral Patol Oral Cir Bucal.

[23] Norheim PW, Dahl BL (1978). Some self-reported symptoms of temporomandibular joint dysfunction in a population in Northern Norway. J Oral Rehabil.

[24] Wanman A, Agerberg G (1986). Headache and dysfunction of the masticatory system in adolescents. Cephalalgia.

[25] Kronn E (1993). The incidence of TMJ dysfunction in patients who have suffered a cervical whiplash injury following a traffic accident. J Orofac Pain.

[26] Choi YS, Choung PH, Moon HS, Kim SG (2002). Temporomandibular disorders in 19-year-old Korean men. J Oral Maxillofac Surg.

[27] Klobas L, Tegelberg A, Axelsson S (2004). Symptoms and signs of temporomandibular disorders in individuals with chronic whiplash-associated disorders. Swed Dent J.

[28] Speculand B, Hughes AO, Goss AN (1984). Role of recent stressful life events experience in the onset of TMJ dysfunction pain. Community Dent Oral Epidemiol.

[29] Auerbach SM, Laskin DM, Frantsve LM, Orr T (2001). Depression, pain, exposure to stressful life events, and long-term outcomes in temporomandibular disorder patients. J Oral Maxillofac Surg.

[30] Filho J, Manzi FR, de Freitas DQ, Boscolo FN, de Almeida SM (2007). Evaluation of temporomandibular joint in stress-free patients. Dentomaxillofac Radiol.

